# Supplementary material for: Optical wireless information transfer with nonlinear micromechanical resonators
Source: Microsyst Nanoeng. 2017 Aug 28;3:17026. doi: 10.1038/micronano.2017.26 (PMC6444992; doi:10.1038/micronano.2017.26)
Supplement: Supplementary Information [file micronano201726-s1.pdf]

Supplementary file

Optical wireless information transfer with nonlinear micromechanical resonators

Joseph A. Boales[1], Farrukh Mateen[2] and Pritiraj Mohanty[1]

Microsystems & Nanoengineering (2017) **3,** 17026; doi:10.1038/micronano.2017.26; Published online: 28 August 2017

TEMPERATURE DEPENDENCE CHARACTERIZATION

Since temperature can have an impact on the measurements taken in this experiment, we characterized the resonance peak of interest as temperature varies, as shown in Supplementary Figure S1a. As the temperature increases, the resonator "softens" and the frequency of the resonance peak decreases at an approximate rate of 16.4 ppm/°C. This measurement was done by heating or cooling the resonator using a Peltier module and measuring the temperature using a thermistor.

After characterizing the location of the resonance peak as a function of temperature, we quantified how much the resonator's temperature increases during each experiment, as shown in Supplementary Figure S1b. The "average laser diode current" refers to the time average of the current. This measurement was done using a 50%-duty-cycle pulse train, so, for example, a 40 mA average current corresponds to a pulse train that peaks 80 mA. After setting the laser diode current at each level, we waited for a period of at least 5 min to ensure that the resonator temperature had reached a steady state. For low laser currents (less than 20 mA), there is no apparent heating in the resonator. Beyond that, the resonator temperature increases approximately linearly with laser current. Based on these measurements, the temperature of the resonator was approximately 1.6 °C above ambient temperature for the results presented in our paper.

FURTHER CHARACTERIZATION OF FIRST ORDER UPPER SIDEBAND

In addition to the sideband characterizations presented in our paper, we performed measurements presented below. In order to successfully transmit data, it is useful to produce the largest sideband amplitude possible, but to avoid noisy regions in the frequency spectrum as much as possible. To assist in choosing a laser modulation frequency, the results in Supplementary Figure S2a were used.

These results show how the sideband size varies with the laser modulation frequency. As the modulation frequency increases, the sideband size decreases approximately exponentially. Evidently, a small modulation frequency should be most effective. However, as approaches the frequency of the carrier signal, the size of the noise increases rapidly, and can hide the sideband at random times. We found that the noise signal was small enough that we could consistently measure the sideband successfully when the modulation frequency was larger than 1 kHz.

In addition, we verified that that the sideband size increases with the size of the modulation signal (Supplementary Figure S2b) as well as with the size of the carrier signal (Supplementary Figure S2c). From these results, it can be seen that the sideband is not measureable until the average laser diode current is at least 30 mA. While the results shown are only for an oscillator power of 0 dBm, this threshold was consistent for other powers as well. Based on these plots, it is obvious that the largest sideband can is obtained by maximizing the LD current and the oscillator driving power.

EFFECTIVE MODAL MASS AND MODAL STIFFNESS

Using COMSOL, the mode shown in Supplementary Figure S3 was found to be the modeshape of the resonance used in our experiments.

Using that modeshape, the effective mass was calculated using

$$m_{\text{eff}} = \int \rho U^2 \mathrm{d}V$$

where m_{eff} is the effective mass, ρ is the material density, and U is the modeshape, normalized such that the maximum displacement is 1. The effective mass was found to be 24.5 ng. The effective modal stiffness can then be found using

$$k_{\text{eff}} = m_{\text{eff}}\omega_0^2 = 14.3\ \text{MN m}^{-1}$$

where k_{eff} is the effective stiffness and ω_0 is the angular resonance frequency.

EFFECTIVE FORCE PRODUCED BY ELECTRODES

To first order, the substrate (silicon and silicon dioxide layers) are assumed to be stationary. Using basic analysis of the AlN layer, we calculate that the location of the center of mass (COM), relative to the grounding electrode, moves according to

$$z_{\text{COM}} = \frac{t_0}{2} + \frac{d_{33}V_0}{2}\sin 2\pi f t$$

where t_0 is the natural thickness of the AlN without applied stresses or fields, d_{33} is the piezoelectric charge constant relating deformation along the thickness to the potential change along the thickness, V_0 is the peak applied voltage, f is the driving frequency, and t is time. Using this, the amplitude of the force applied to the AlN by the substrate is

$$F_{\text{eff}} = m\ddot{z}_{\text{COM,max}} = 2\pi^2 m d_{33} V_0 f^2$$

where m is the mass of the material expanding and contracting due to the applied potential. For our resonator, this comes to approximately 342 µN. Normalizing this by the mode shape gives us an effective force of 10.6 µN.

[1]Department of Physics, Boston University, 590 Commonwealth Avenue, Boston, MA 02215, USA and [2]Department of Mechanical and Aerospace Engineering, Boston University, 110 Cummington Street, Boston, MA 02215, USA
Correspondence: Mohanty Pritiraj (mohanty@physics.bu.edu)

ESTIMATION OF FORCE PRODUCED BY RADIATION PRESSURE

The radiation pressure produced by an electromagnetic wave can be written as

$$P_{\mathrm{rad}} = (R + 1) I_{\mathrm{rad}} / \mathrm{c}, \tag{S1}$$

where R is the reflectance of the material on which the radiation is incident, I_{rad} is the intensity of the wave, and c is the speed of light. Supplementary Equation S1 assumes that all incident radiation is either absorbed or reflected from the surface, and that none is transmitted through the resonator. The top surface of our MEMS resonator is primarily covered with molybdenum, which as a reflectivity near 60% at wavelength 520 nm.

The specific laser diode that we used in this experiment has an output of 15.0 mW when the current is 141.6 mA. The beam passes through a collimator, which produces a spot with diameter, *2 w*, of 1.48 mm (using the $1/e^2$ method) and which diverges at 0.029 degrees. At the surface of the resonator which is about 30 cm from the collimator, the spot size is 1.78 mm.

The beam is estimated to have a Gaussian profile, so 86.47% of the optical power is deposited within the 1.78-mm spot. It is difficult to verify that oscillator is precisely in the center of the spot, so we average the power over the spot size (so as to not overestimate the force) to find an average intensity of 5190 $\mathrm{Wm^{-2}}$. Inserting this into Supplementary Equation S1, and multiplying it by the 270-by-96 micron top surface area of the resonator, we find an approximate radiation force of 0.718 pN. Note that the area used in calculating the modeshape should be normalized by the mode shape. However, since we use the average laser intensity instead of the maximum, we assert that these errors approximately cancel. Further, this number is used as an order-of-magnitude estimate for comparison purposes only; the exact size of the force is not critical to the results presented in the paper.

DUFFING EQUATION FAST FOURIER TRANSFORM

To numerically verify that upper and lower sidebands can be produced by driving the duffing resonator with a large signal at resonance and a small non-resonant frequency to produce a sideband, we performed numerical simulations using Wolfram Mathematica. The first-order lower and upper sidebands are in Supplementary Figure S4. Simulations were performed using a resonance frequency normalized to 1, a Q of 1000, and softening k_3 value of − 1. The resonant signal is 10 times larger than the non-resonant signal.

The code used to generate the data in the plot above is below.

```
ClearAll["Global`*"]
Fs = 200; (* Sampling Rate in Hz *)
T = 10000; (* Sampling period in s *)
L = T*Fs; (* Number of points in signal table*)
stepSize = 0.001;
Q = 1000;
f0 = 1;
f1 = f0;
f2 = 0.02;
ω0 = 2π f0;
ω1 = 2π f1;
ω2 = 2π f2;
NDSolve[{x''[t]+ ω0^2 x[t]+ω0/Q x'[t]–x[t]^3 = = 10Cos[ω1 t+π]+(1
+SquareWave[f2 t])/2,x[0] = = 0,x'[0] = = 0},x[t],{t,0,T},MaxStep-
Size- > stepSize];
f[t_]: = Evaluate[x[t]/.%]
times = Table[N[n/Fs],{n,L}];
data = Table[First[f[n/Fs]],{n,L}];
newData = Table[{Part[times,n],Part[data,n]},{n,L}];
ListLinePlot[newData,PlotRange- > All]
response = Abs[Fourier[data]];
freqs = Table[N[n Fs/L],{n,L/2}];
freqResponse = Table[{Part[freqs,n],(Part[response,n]+Part
[response,L+1–n])},{n,L/2}];
```

The results may be plotted using a list line plot.

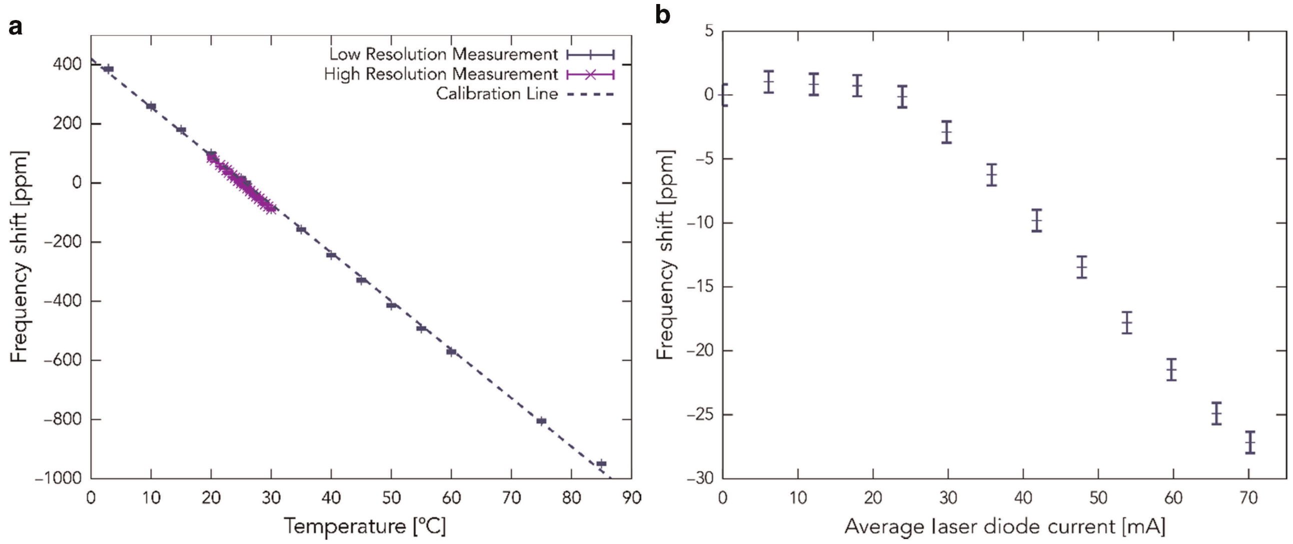

Figure S1 (**a**) Frequency shift of 120.4 MHz resonance peak as a function of temperature. The shift is measured in parts per million of the frequency at 25 °C. (**b**) Shift of the 120.4 MHz resonance peak as the laser diode current (power) is increased. As the current increases, the steady state frequency is decreased, indicating heating. Beyond 20 mA, the resonance peak shifts at an approximate rate of -0.6 ppm mA^{-1}, indicating a steady-state temperature increase of 0.04 °C mA^{-1}.

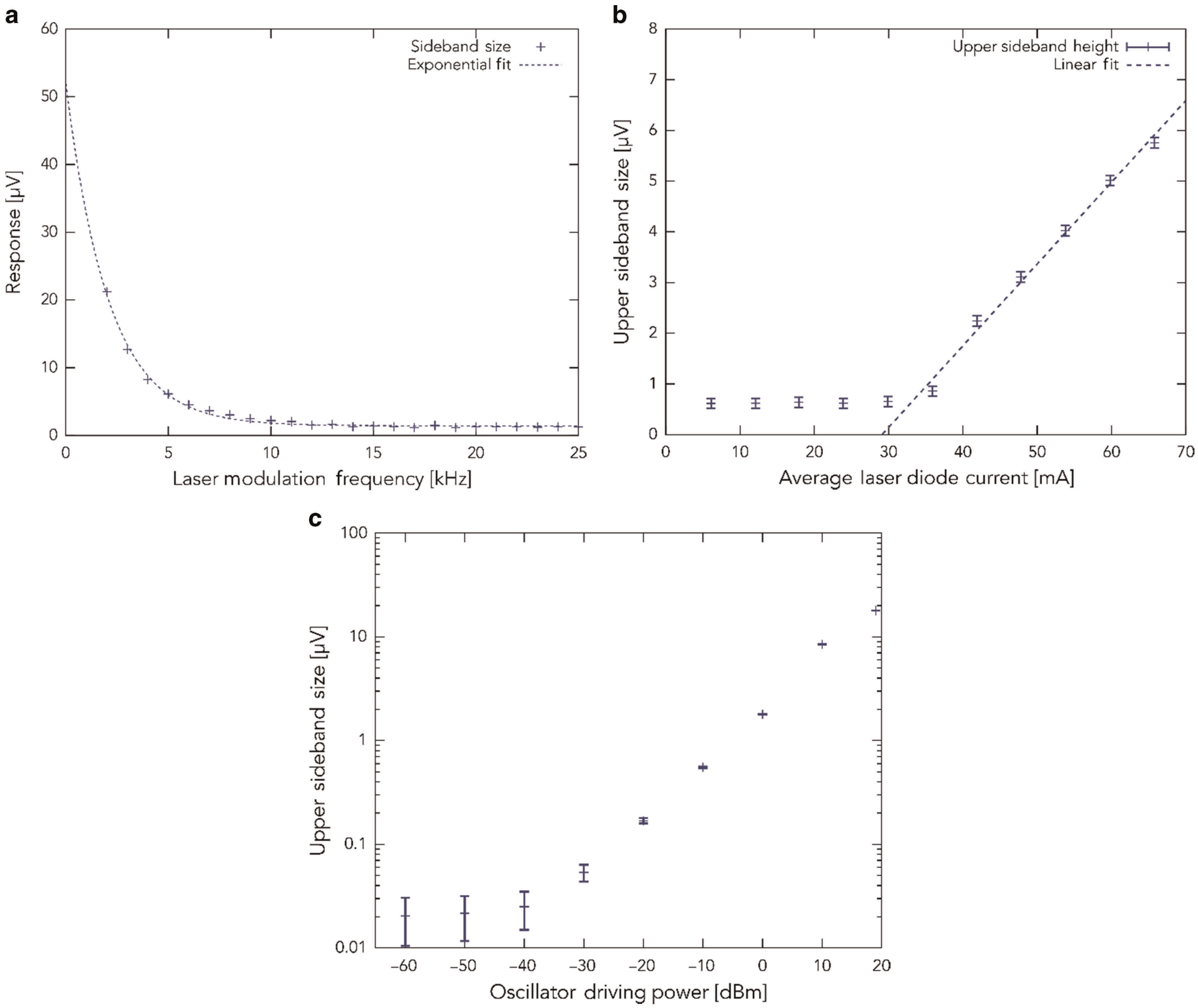

Figure S2 (**a**) Upper sideband size as a function of laser modulation frequency for 19 dBm oscillator driving and 70 mA average LD current. As the frequency increases, the sideband size exponentially decreases. (**b**) Sideband size as function of laser diode current for 0 dBm oscillator driving. Near 30 mA, the LD power is high enough to begin producing measureable sidebands. After that the sideband size increases linearly with LD current. (**c**) Sideband size as function of oscillator driving power. As the power to the oscillator is increased and its response becomes increasingly nonlinear, the sideband grows.

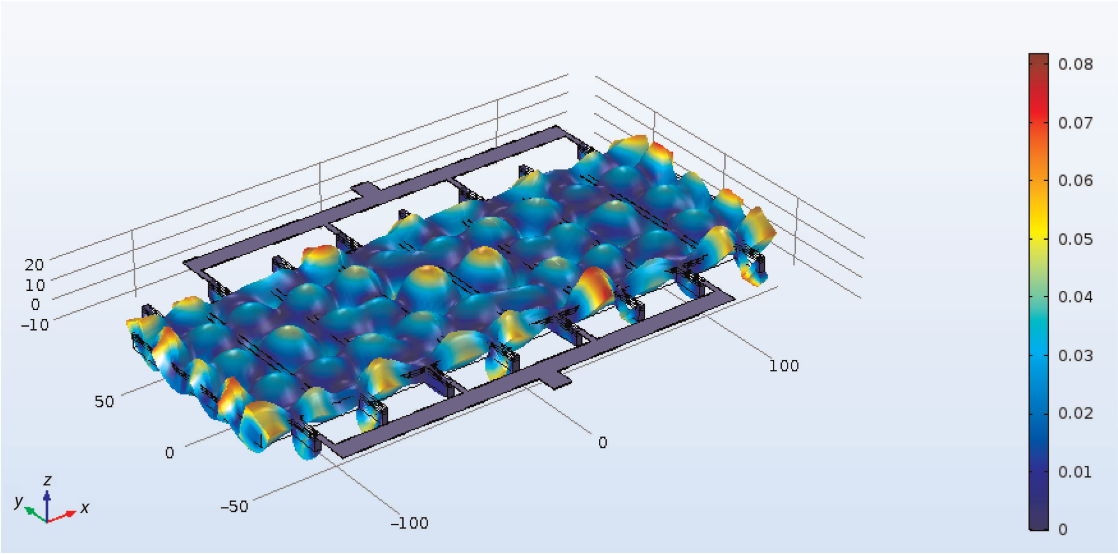

Figure S3 Numerically-identified modeshape used in wireless data transfer experiments. Color scale is arbitrary, and length scale is in microns.

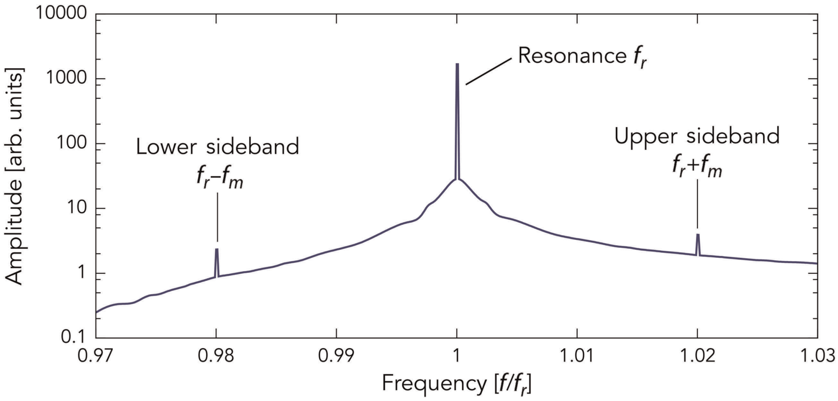

Figure S4 Frequency spectrum numerically generated using a Duffing resonator driven at resonance and at a smaller modulation frequency. The amplitude is on a logarithmic scale so the sidebands can be seen in the same plot as the resonance.
